# Stress in Medical Students: PRIMES, an Italian, Multicenter Cross-Sectional Study

**DOI:** 10.3390/ijerph19095010

**Published:** 2022-04-20

**Authors:** Paolo Leombruni, Alessio Corradi, Giuseppina Lo Moro, Anna Acampora, Antonella Agodi, Daniele Celotto, Maria Chironna, Silvia Cocchio, Vincenza Cofini, Marcello Mario D’Errico, Carolina Marzuillo, Maria Pavia, Vincenzo Restivo, Licia Veronesi, Maria Rosaria Gualano, Fabrizio Bert, Roberta Siliquini, on behalf of the PRIMES Collaborating Group

**Affiliations:** 1 Clinical Psychology Unit, Department of Neurosciences, University of Torino, 10126 Torino, Italy; paolo.leombruni@unito.it; 2 Department of Sciences of Public Health and Pediatrics, University of Torino, 10126 Torino, Italy; giuseppina.lomoro@unito.it (G.L.M.); mariarosaria.gualano@unito.it (M.R.G.); fabrizio.bert@unito.it (F.B.); roberta.siliquini@unito.it (R.S.); 3 Sezione di Igiene, Istituto di Sanità Pubblica, Università Cattolica del Sacro Cuore, 00168 Roma, Italy; annina.acampora@gmail.com; 4 Department of Medical and Surgical Sciences and Advanced Technologies “GF Ingrassia”, University of Catania, 95124 Catania, Italy; agodia@unict.it; 5 Department of Medicine, University of Udine, 33100 Udine, Italy; daniele.celotto@asufc.sanita.fvg.it; 6 Department of Biomedical Sciences and Human Oncology, University of Bari—Aldo Moro, 70121 Bari, Italy; maria.chironna@uniba.it; 7 Department of Cardiac, Thoracic, Vascular Sciences and Public Health, University of Padova, 35128 Padova, Italy; silvia.cocchio@unipd.it; 8 Biostatistics and Epidemiology Unit, Department of Life, Health and Environmental Sciences, University of L’Aquila, 67100 L’Aquila, Italy; vincenza.cofini@univaq.it; 9 Department of Biomedical Sciences and Public Health, Polytechnic University of Marche, 60020 Ancona, Italy; derrico@univpm.it; 10 Department of Public Health and Infectious Diseases, Sapienza University of Roma, 00185 Roma, Italy; carolina.marzuillo@uniroma1.it; 11 Department of Health Sciences, University “Magna Graecia” of Catanzaro, 88100 Catanzaro, Italy; pavia@unicz.it; 12 Department of Science for Health Promotion and Mother to Child Care “G. D’Alessandro”, University of Palermo, 90217 Palermo, Italy; vincenzo.restivo@unipa.it; 13 Department of Medicine and Surgery, University of Parma, 43121 Parma, Italy; licia.veronesi@unipr.it; 14 Department of Quality and Safety of Care, Azienda Ospedaliero-Universitaria City of Health and Science of Torino, 10126 Torino, Italy

**Keywords:** psychological distress, stress, psychological, schools, medical, students, medical, cross-sectional studies

## Abstract

Medical students (MSs) are healthcare workers and must also cope with education-related stressors. This study aims to assess factors associated with perceived stress in Italian MSs. A cross-sectional study was conducted in 12 Italian medical schools (MSCs) in November 2018. A questionnaire assessed socio-demographic characteristics, habits, opinions about MSC, and concerns about the future. Stress was assessed with the PSS-10. Descriptive and univariable tests were performed. A linear model was fitted to find associations of the PSS-10 score with characteristics. There were 2513 collected questionnaires. Median PSS-10 score was 18 (IQR 11). Median age was 22 (IQR 4) and 61% of the sample was female. Female gender, being part of a sexual minority, poor financial situation, competitive atmosphere, having hobbies, resting, and sleeping hindered by MSC were characteristics associated with higher PSS-10 scores. Current relationship, good family relationship, and no concerns about the future were protective factors. Being part of sexual minorities had greater effects in students not living away from home, while in the other group it was not having satisfying friendships. Medical students suffer higher levels of stress than the general population, and many MSC stressors are associated. Living away from home modifies risk and protective factors, offering the possibility to tailor group-specific interventions.

## 1. Introduction

Stress is the body’s response to pressure from life events, especially those that are unexpected and exceed personal coping strategies or directly threaten the ego [1]. It is well known that chronic stress not only impairs cognitive functions such as memory and cognition [2,3], but also immune, cardiovascular, gastrointestinal, and endocrine systems [3]. One of the most well-studied kinds of stress is work-related stress. Some categories of workers, such as healthcare workers, are at higher risk of developing high stress and burnout [4,5].

Additionally, medical students (MSs) share this higher susceptibility to stress [6,7], with some data suggesting that half of all MSs may be affected by burnout during their medical education [8], and this is perhaps an underestimation [9]. MSs are particularly exposed to multiple stressors, a diverse combination of academic deadlines [10], and daily experiences of health assistance-related challenges [11]. Finally, MSs frequently face mistreatment, which further increases the risk of stress and burnout [12,13].

Stigma may prevent MSs from asking for help, due to them being fearful of exposure to colleagues’ judgment [14]. The non-supportive climate of competition, rising with career progression, causes both stress building and shame of vulnerabilities [15].

Stress is associated with decreased satisfaction, thoughts of dropping out, and suicidal ideations, in a vicious circle that affects academic performance which causes more stress in turn [16].

Individual risk factors were described, such as female gender [17] or chronic disease [18]; furthermore, medical students are often ambitious and hard-working people [19], characteristics associated with a higher risk of developing stress [20]. However, stress is also related to environmental factors [6].

Stress in European MSs is described in the scientific literature [9], but to our knowledge no Italian nationwide data are available. In Italy, every aspiring MS must undergo a national test to compete with other participants for a limited number of places. Furthermore, students come from all over the country, but medical schools (MSCs) are in big cities, and in limited numbers (41 MSCs). Winners choose what university they will study in, if there are still places, and higher-ranking students choose first. Thus, students might need to move away from home [21] to reach the university of choice, or in the case of lower scores, students might need to move away from home to enrol at all in a medical school. For this reason, the stress acquired because of the medical school (MSC) per se seems to be exacerbated by new commitments such as shopping for food and travel [22]. Indeed, some studies have shown that medical students who have their accommodation away from home may have a higher risk of stress [23,24,25]; however, such evidence is limited, and it mostly refers to international contexts very different from the Italian one. Once the student is enrolled, MSCs last 6 years, with the first three years focusing on preclinical subjects and the last three years on clinical ones. In most MSCs, the first internship occurs in 4th year. The grade system uses a 30-point scale, and the grade average is used to calculate the mark of the degree. These scores give additional points for the specialty school test [26], another nationwide test with limited places. These factors contribute to building up competition and to the importance acknowledged by students and families of both MSC grades and mark of the degree.

Even if the definition of stress is evolving and the “historic” division between eustress and distress has been questioned [27], effects of stress on well-being are of undoubtful weight [3].

Therefore, this study aims to assess the spread of perceived stress among Italian MSs, to find and weigh possible risk and protective factors associated with this burden. Furthermore, this study aims to define the importance of the living (or not living) away from home status as a risk factor associated with developing stress in medical students, and to identify possible risk and protective factors which are specific to this characteristic, to better tailor and offer preventive interventions and treatments.

## 2. Materials and Methods

### 2.1. Study Design

A multicenter cross-sectional study named Psychosocial Report in Italian MEdical Students (PRIMES) [28] was conducted on medical students attending 12 MSCs (29.27% of Italian MSCs [29]) spread over the country (4 in northern, 3 in central, and 5 in southern Italy (ISTAT Istituto Nazionale di Statistica, [30])), representing 29.27% of the 41 Italian medical schools. Students were enrolled by convenience sampling in 1st, 4th, and 6th year of the course, with an eligible population of approximately 9000 participants. Data were collected far from exams (November 2018). A minimum sample size of 383 was calculated with Raosoft software and with the following parameters: 5% margin of error, 95% confidence level, 50% response distribution, population of 78,101 (MSs in 2017 [29]).

Participants were required to read and sign an informed consent prior to questionnaire filling. All procedures performed were in accordance with the 1964 Helsinki Declaration and its later amendments. Protocol was approved by the Ethics Committee of the University of Turin.

A self-administered questionnaire was offered in-class to enrolled medical students.

### 2.2. The Questionnaire

A self-administered questionnaire was developed, with a custom 30-item socio-demographic part (retrievable in Supplementary Materials) and a section whose purpose was to assess stress, the Perceived Stress Scale, 10-item version (PSS-10) [31].

Items of the socio-demographic section were meant to describe possible risk and protective factors described in the literature. Therefore, participants were asked their gender, age, nationality, family history of psychiatric disorders and suicides, personal chronic diseases, economic situation, employment condition [6], distance from home [22], people living with them, perceived family cohesion and relationship status [6,32,33,34,35], sexual orientation [36], practicing sport [37] or having a hobby [32], year of medical school attended, current performance in medical studies [32], perceived climate among classmates, and presence of satisfying friendships among them [6,11,34]. Furthermore, students were asked if they were feeling that MSC was hindering specific activities (e.g., sleeping, practicing sport) in their lives [38], what motivations were behind their university choice (e.g., helping others, intellectual curiosity) [39], and their concerns about the future [22]. More than one answer was accepted for these latter questions.

Since MSs undertreat their psychological or psychiatric conditions [14], the use of psychiatric drugs and stimulants was assessed [40].

The second section’s purpose was to assess perceived stress and stress symptoms. The PSS-10 was used. The PSS-10 is one of the most employed self-administered scales with this purpose [31], and has already been validated on a sample of university students [41]. PSS-10 items investigate the amount of life unpredictability and how much the interviewee was able to cope with it in the past 30 days. Questions are broad and fit to any population. Every item is a Likert scale from 0 to 4 but scores of some questions must be reversed (e.g., 0 = 4, 1 = 3). Therefore, total PSS-10 score (the main outcome of this study) ranges from 0 to 40 and a higher score correlates with higher perceived stress. PSS-10 score was treated as a continuous variable as there is still a lack of strong evidence of validated cut-offs. However, since some cut-offs have been adopted, for the purpose of comparability, proportions in “low risk of stress”, “medium risk of stress”, and “high risk of stress” groups are reported (0–13, 14–26, 27–40 total PSS-10 score, respectively) [42].

### 2.3. Statistical Analysis

Descriptive analyses were performed for all variables. Age and PSS-10 score were treated as continuous variables and are described with medians and interquartile ranges (IQRs) because of non-normal distribution (Shapiro–Wilk test). Categorical variables are described as frequencies and percentages. The same descriptive analyses were performed by stratifying the sample for away from home status: not living away from home (NLAfH) and living away from home (LAfH) subgroups were created. Then, differences between variables in the subgroups were assessed with Mann–Whitney U tests (continuous variables) and chi-square tests (categorical variables). Categorical variables were further analyzed by evaluating adjusted residuals to define pairwise differences. Median PSS-10 score was calculated for every subgroup of characteristics (e.g., males, females) and stratifying for away from home status. Statistically significant differences among subgroups were assessed via Mann–Whitney U tests and Kruskal–Wallis tests.

We developed a multivariable linear regression model to weigh associations of characteristics with perceived stress. First, suitable characteristics were entered in the model, and then selected via a backward stepwise method. Cook’s distance was then used to remove outliers. The final regression model was performed on this selected sample and with these selected characteristics, both on the whole sample and stratifying for away from home status. This final model was checked for collinearity between variables. Results are expressed as unstandardized coefficients (B) and 95% confidence intervals (95% CIs). SPSS software (version 25) was used and a two-tailed *p*-value < 0.05 was considered. Missing values were excluded pairwise for descriptive analyses, listwise for others.

## 3. Results

### 3.1. Characteristics of the Sample

A total of 2513 questionnaires were collected (28% response rate), but only 2455 students (97.69% of these) completed the PSS-10 section. PSS-10 median score was 18, with a high spread of results (IQR 11), while the mean was 18.56 (7.79 SD). Looking at categories, 27.9% had a “low-risk”, 55.2% a “medium-risk”, and 16.9% a “high-risk” score (data not presented in table).

Median age was of 22 years (IQR 4), and 61% of the sample was female. Years of the course were evenly represented, with 41% of respondents in 1st year, 30% in 4th year, and 29% in 6th year. A “Good” financial situation was reported by 90% of respondents, with 4% of the sample declaring having a job because of economic need. Regarding sport, 43% of the sample reported more than 90′ of physical activity per week, but 28% reported none. Sixteen percent of the sample described the medical class atmosphere as “Competitive and hostile”, and a minor part (5%) reported no satisfying friendship with classmates. In MSs’ opinion, MSC hinders having hobbies above all (53% of the sample), then resting (48%). MSC was chosen because of the need to help those who suffer (54.4% of the sample) and intellectual curiosity (45%). Finally, 47% of the sample felt not good enough for the medical profession, and 46% had concerns about limited post-degree opportunities (work, specialty school). Five percent of the sample reported use of stimulating drugs. Full descriptive data are available in Table 1.

Regarding living away from home status, the majority (60%) of the sample reported living away from home. No differences were found in PSS-10 score medians (18) and IQRs (11) among the two subgroups. The subgroups had similar distribution for most of the characteristics, with notable exceptions: LAfH students were more frequently single (51% vs. 45%) and were living much less frequently with their families (22% vs. 94%) but reported more often an “Excellent” evaluation of the relationship (55% vs. 46%). Finally, 37.5% of LAfH students declared more than 90 min of sport per week, compared to 51% of the NLAfH subgroup. A higher proportion of LAfH students reported that MSC hinders having hobbies (54.5% vs. 50%). Full data about the comparison are reported in Table 1.

### 3.2. Perceived Stress—Associated Characteristics

Table 2 shows PSS-10 median scores and IQRs for every characteristic subset (e.g., males and females), in the full sample and in LAfH and NLAfH subgroups.

Associations between PSS-10 score and characteristics were similar in LAFH and NLAfH subgroups, with notable exceptions. A higher PSS-10 score (23) was found in NLAfH students with a poor evaluation of the relationship with their family than in students with a good or excellent evaluation, and this association remained in LAfH students (21). Conversely, an excellent family relationship evaluation was associated with lower scores of the PSS-10 in NLAfH students. Having a job because of need was associated with higher PSS-10 scores in the NLAfH subgroup (24 vs. 20). LAfH students who did not play sport at all reported higher scores than the other subgroup, and the difference with students reporting sport activities was statistically significant in this subgroup (*p* < 0.001).

The results of multivariable linear regression models are reported in Table 3. Age was associated with higher stress level, but only in the LAfH subgroup (*p* = 0.006). Female gender was associated with stress in every subgroup, while studying in central and southern Italy, compared to northern Italy, was associated with higher stress more strongly in NLAfH students. Students attending 6th year reported lower PSS-10 scores, but only in the LAfH subgroup. The same subgroup reported higher scores if a chronic disease was present. A current relationship was associated with lower levels of perceived stress. A good or higher family relationship evaluation, having hobbies, intellectual curiosity as a motivation to enrol in MSC, and not having concerns about the future were all characteristics associated with lower PSS-10 scores in both subgroups. Conversely, being part of a sexual minority, a poor financial situation, working because of need, a negative opinion on the MSC choice, thinking the atmosphere in classes is competitive and hostile and not having satisfying friendships with classmates, feeling that MSC prevents having hobbies, sleeping adequately, and resting, and having concerns about the future were all characteristics associated with a higher level of perceived stress, although with notable differences in effect size among subgroups. Being part of a sexual minority had a greater effect in NLAfH students, while not having satisfying friendships had the biggest effect on PSS-10 scores of all assessed characteristics among LAfH students.

## 4. Discussion

The aim of this study was to investigate spread of perceived stress in Italian medical students, to assess and weigh possible risk and protective factors associated with perceived stress. Overall, 55.2% and 16.9% of the sample reported, respectively, medium risk and high risk of perceived stress. Given that there is no standardized test widely used in medical students [7] to measure stress, a direct comparison with other countries is difficult, as it must be limited to other studies implementing PSS-10. A Russian study reported 69.1% and 4.9% in the same medium-risk and high-risk categories [43]. A recent Polish study reported higher stress levels than those of the present work, with a mean of 22.78 in Polish students [44], while in Saudi Arabia lower levels of perceived stress were reported [45]. A Chinese study reported a slightly lower mean (18.06) in a sample of dental school students [46]. Interestingly, an Italian investigation on non-medical students reported a lower prevalence of high risk of perceived stress (10.7%), thus suggesting that the course of study might influence the development of a high level of stress [47].

Even if living away from home is a known risk factor for stress [48,49], this was not evident in our sample. However, it can be hypothesized that differences in risk and protective factors, both in terms of prevalence and weight, can explain this finding.

Regarding factors associated with higher levels of stress, female gender was one of the most important characteristics, as already reported by other authors [6]. Interestingly, age (adjusted for, among others, year of university currently attending) was associated with higher stress levels only in LAfH students. A possible explanation may be that these students have higher living expenses and thus may have more guilty feelings asking for resources from their families for longer periods of time. Another explanation could be that society and medical student peers expect them to already be working (social and peer pressure). In the same perspective, LAfH students found relief in attending the last year of university, while the same was not true in the NLAfH subgroup. At the same time, while having a chronic condition is a risk factor for stress [6], in our sample only LAfH students had a higher level of stress in the presence of a chronic condition. In a recent work [50], it has been demonstrated that LAfH university students struggle to find proper care and choose to self-treat, even if in Italy the healthcare system is free and it is not difficult to obtain general practitioner assistance.

Involvement in a relationship is a well-known protective factor for psychological well-being, already demonstrated in medical students [6]. In this study, the statistical significance of this association was demonstrated only in NLAfH students, but a mild, non-significant effect (*p* = 0.117) was also found in LAfH students. However, a clear and statistically significant effect was found for non-heterosexual sexual attraction, and the size of the effect was greater in NLAfH students. Being part of a “sexual minority”, in a country that still applies stigmas and discrimination [51], has widely described effects on psychological well-being [36]. However, the results of this study suggest that non-heterosexual students who were forced (or who chose) to move from their home still show a higher level of stress, but to a lesser extent compared to those students who, living in their home cities, probably still have to face stigma from society or even their families.

Unsurprisingly, the effect of the quality of the relationship with family, that has the role of a social support [6], was greater in NLAfH students. However, it must be noted that students living with their families were less prone to define their relationship as “Excellent”.

Regarding financial situation, adjusted regression showed that working was not associated with stress per se, but because of the bad financial situation that often required MSs to work. A bad financial situation has already been associated with higher stress levels in medical students [52].

Regarding free time, surprisingly, sport was not associated with lower level of stress in the adjusted model, but almost obtained statistical significance in the whole sample analysis (*p* = 0.051). Since sport has widely known positive effects on mental health [28,53], this finding should be deepened by further studies. However, the mere fact that 31.5% of LAfH students and 23.5% of NLAfH ones do not play sport at all could contribute to the high PSS-10 scores measured. In the same context, feeling that MSC prevents having hobbies, sleeping, and resting was associated with higher stress levels, and this finding was true for both subgroups.

Other factors associated with higher PSS-10 score were social ones. Thinking that the atmosphere in the medical class is competitive and hostile and not having satisfying friendships were independently associated with higher stress. However, the latter reached statistical significance (*p* < 0.001) in the LAfH subgroup only, suggesting that these students do not have any local social networks that can compensate for the bad relationship status with other medical students [54].

Finally, lack of motivation, i.e., negatively considering the choice of becoming an MS, was associated with higher PSS-10 score in both subgroups. Medical students having a lower level of perceived stress reported the future as stimulating, but it was higher if they felt not good enough for the profession. Interestingly, these concerns had a greater effect on stress in the LAfH students, possibly suggesting that these students fear the end of university more, with the risk of returning to their home cities and possibly being cut away from their new social network, in the case of a bad result on the specialty school national test.

This work has several limitations and strengths. First, the sampling was convenient and not randomized. However, an acceptable representativeness was achieved with wide sampling over the entire country of a considerable number of universities. Second, findings are associations that cannot be considered as causal links, given the cross-sectional study design. Still, this study had the aim to assess the presence of well-known risk factors for stress in a specific population and in specific subgroups, and it is the first multicenter study assessing stress prevalence in Italian medical students.

## 5. Conclusions

This study assessed stress and characteristics associated with stress in a large sample of medical students, furtherly stratifying the analysis by living status. These new findings, which further studies could generalize to the whole population of university students, seem to suggest that these two subgroups share many risk factors for stress, but others are specific for each subgroup. Thus, stakeholders interested in MSs’ health should offer specific and tailored interventions, accounting for those differences.

## Figures and Tables

**Table 1:** Characteristics of the sample.

Characteristic		All Students n = 2513		NLAfH 39.6%		LAfH 60.4%		*p*
**Age**		22	(4)	22	(4)	22	(4)	0.539
**PSS-10 score**		18	(11)	18	(11)	18	(11)	0.756
**Gender**	*Male*	38.6		38.9		38.5		0.824
	*Female*	61.4		61.0		61.4		
**University location**	*Northern Italy*	39.6		41.2		38.6		0.284
	*Central Italy*	22.2		20.7		23.1		
	*Southern Italy*	38.1		38.0		38.1		
**Year of university**	***1st***	41.2		41.7		40.8		**0.046**
	***4th***	**29.7**		**27.1**	**ᵇ**	**31.4**	**ᵃ**	
	***6th***	29.0		31.1		27.6		
**Chronic disease †**		7.1		7.0		7.1		0.928
**Currently in a relationship**	***Single***	**48.4**		**44.7**	**ᵇ**	**50.9**	**ᵃ**	**0.002**
	***Involved***	**51.5**		**55.2**	**ᵃ**	**49.0**	**ᵇ**	
**Being part of a sexual minority**	*No*	86.1		84.8		87.0		0.123
	*Yes*	13.9		15.2		13.0		
**Current housemates**	***No one***	**4.6**		**1.4**	**ᵃ**	**6.7**	**ᵇ**	**<0.001**
	***Partner/Friends***	**45.1**		**4.7**	**ᵃ**	**71.5**	**ᵇ**	
	***Family***	**50.3**		**93.9**	**ᵇ**	**21.8**	**ᵃ**	
**Family history of mental illness †**		23.8		23.4		24.1		0.696
**Family history of suicide †**		5.8		5.6		6.0		0.682
**Family relationship evaluation**	*Poor/excessive*	10.9		12.4		10.0		**<0.001**
	***Good***	**37.5**		**41.9**	**ᵃ**	**34.6**	**ᵇ**	
	***Excellent***	**51.4**		**45.6**	**ᵇ**	**55.3**	**ᵃ**	
**Financial situation**	*Good*	89.7		89.0		90.1		0.393
	*Poor*	10.3		10.9		9.8		
**Employment situation**	*Have a job because of need*	4.4		5.3		96.3		0.059
	*Do not have a job/Have a job but do not need it*	95.6		94.7		3.7		
**Having a hobby †**		69.8		70.6		69.1		0.419
**Playing sport**	***No***	**28.3**		**23.5**	**ᵇ**	**31.5**	**ᵃ**	**<0.001**
	***Less than 90′ a week***	**28.8**		**25.5**	**ᵇ**	**31.0**	**ᵃ**	
	***More than 90′ a week***	**42.8**		**51.0**	**ᵃ**	**37.5**	**ᵇ**	
**Current opinion on the medical school choice**	*Positive*	79.2		80.4		78.4		0.217
	*Negative/No opinion*	20.8		19.5		21.5		
**Current opinion on medical class atmosphere**	*Friendly/Stimulating/No opinion*	83.8		84.3		83.4		0.553
	*Competitive and hostile*	16.1		15.6		16.5		
**Satisfying friendships with classmates**	*Yes/Not yet*	94.5		94.6		94.5		0.948
	*No*	5.4		5.3		5.4		
**Feeling that medical school prevents ‡**	*Playing sport*	45.1		43.1		46.3		0.117
	***Having hobbies***	**52.7**		**49.9**	**ᵇ**	**54.5**	**ᵃ**	**0.025**
	*Sleeping adequately*	40.4		40.8		40.1		0.721
	*Seeing friends*	42.9		44.2		42.1		0.319
	*Resting*	48.3		48.2		48.3		0.926
**Motives underlying medical school choice ‡**	*High gain opportunities*	10.0		10.3		9.87		0.717
	*Employment opportunities/Social prestige*	29.3		29.0		29.5		0.775
	*Personal/Family history of disease*	16.6		16.1		17.0		0.571
	*Acquaintance/Relative MD influence*	6.4		7.6		5.7		0.060
	*Helping those who suffer*	51.4		52.3		50.9		0.490
	*Interest in human relationships*	36.8		35.8		37.5		0.412
	*Intellectual curiosity*	45.0		45.3		44.8		0.792
	*Imposition by parents/relatives*	0.5		0.8		0.4		0.175
**Concerns about the future ‡**	*No, future is stimulating*	24.3		25.6		23.4		0.226
	*No, not thinking about future*	6.3		6.6		6.1		0.614
	*Yes, not feeling good enough for the profession*	46.7		46.3		46.9		0.753
	*Yes, about the specialty choice*	20.8		21.8		20.2		0.331
	*Yes, about limited work/specialty opportunities*	46.4		45.9		46.7		0.677
**Stimulating drugs**		4.9		5.0		4.8		0.850

Figures are percentages or medians (IQRs). NLAfH Not Living Away from Home, LAfH Living Away from Home † Yes/no answers. Yes answer prevalence is reported. ‡ More than one answer was possible. ᵃ Adjusted residual > 2.00. ᵇ Adjusted residual < 2.00.

**Table 2:** PSS-10 median score per characteristic per living status.

Characteristic	All Students				NLAfH				LAfH			
	Median		IQR		Median		IQR		Median		IQR	
**Gender**				**<0.001**				**<0.001**				**<0.001**
***Male***	**15**		**10**		**15**		**10**		**16**		**10**	
***Female***	**20**		**11**		**20**		**11**		**20**		**12**	
**University location**				**0.005**				**0.331**				**0.005**
***Northern Italy***	**18**	**ᵃ**	**11**		**18**		**11**		**18**	**ᵃ**	**11**	
***Central Italy***	**19**	**ᵇ**	**13**		**19**		**14**		**19**	**ᵇ**	**12**	
***Southern Italy***	**18**	**ᵃ^,^ᵇ**	**11**		**18**		**9**		**18**	**ᵃ^,^ᵇ**	**11**	
**Year of university**				**<0.001**				**<0.001**				**<0.001**
***1st***	**16**	**ᵃ**	**10**		**15**	**ᵃ**	**10.75**		**16**	**ᵃ**	**10**	
***4th***	**20**	**ᵇ**	**12**		**20**	**ᵇ**	**11**		**20**	**ᵇ**	**12**	
***6th***	**20**	**ᵇ**	**11**		**20**	**ᵇ**	**11**		**19**	**ᵇ**	**10.5**	
**Chronic disease**				**<0.001**				**0.008**				**0.001**
***No***	**18**		**11**		**18**		**11**		**18**		**11**	
***Yes***	**21**		**10.75**		**21**		**16**		**21**		**8.25**	
**Currently in a relationship**				0.728				0.158				0.505
***Single***	18		11		18		10		18		11	
***Involved***	18		11.5		18		11		18		12	
**Being part of a sexual minority**				**<0.001**				**<0.001**				**0.001**
***No***	**18**		**12**		**18**		**11**		**18**		**11**	
***Yes***	**20**		**11**		**20**		**11**		**20.5**		**11.75**	
**Current housemates**				0.174				0.230				0.255
***No one***	17		12		20		10		17		12	
***Partner/Friends***	19		11		21		10.5		18		11	
***Family***	18		11		18		11		18		11	
**Family history of mental illness**				**<0.001**				**<0.001**				**<0.001**
***No***	**18**		**11**		**18**		**11**		**18**		**12**	
***Yes***	**20**		**11**		**20**		**12**		**20**		**11**	
**Family history of suicide**				**<0.001**				**0.030**				**0.001**
***No***	**18**		**11**		**18**		**11**		**18**		**11**	
***Yes***	**20**		**12**		**20**		**11**		**20.5**		**12**	
**Family relationship evaluation**				**<0.001**				**<0.001**				**<0.001**
***Poor/Excessive***	**21**	**^c^**	**11**		**23**	**^c^**	**11**		**21**	**ᵇ**	**10**	
***Good***	**20**	**ᵇ**	**11**		**19**	**ᵇ**	**10**		**20**	**ᵇ**	**12**	
***Excellent***	**16**	**ᵃ**	**11**		**16**	**ᵃ**	**12**		**17**	**ᵃ**	**10**	
**Employment situation**				**<0.001**				**<0.001**				**0.068**
***Have a job because of need***	**22**		**12**		**24**		**9.5**		**20**		**13**	
***Do not have a job/Have a job but do not need it***	**18**		**11**		**18**		**10**		**18**		**11**	
**Financial situation**				**<0.001**				**<0.001**				**<0.001**
***Good***	**18**		**11**		**18**		**11**		**18**		**10**	
***Poor***	**23**		**11**		**23**		**9**		**23**		**12**	
**Having a hobby**				**<0.001**				**<0.001**				**<0.001**
***No***	**21**		**12**		**21**		**12**		**20**		**12**	
***Yes***	**17**		**11**		**17**		**10**		**17**		**11**	
**Playing sport**				**<0.001**				**0.278**				**<0.001**
***No***	**19**	**ᵇ**	**11**		**19**		**11**		**20**	**ᵇ**	**12**	
***Less than 90′ a week***	**18**	**ᵃ^,^ᵇ**	**11**		**19**		**10**		**18**	**ᵃ**	**11**	
***More than 90′ a week***	**18**	**ᵃ**	**11**		**18**		**12**		**17**	**ᵃ**	**11**	
**Current opinion on the medical school choice**				**<0.001**				**<0.001**				**<0.001**
***Positive***	**17**		**10**		**17**		**10**		**17**		**10**	
***Negative/No opinion***	**23**		**11**		**23**		**10.5**		**23**		**11**	
**Current opinion on medical class atmosphere**				**<0.001**				**<0.001**				**<0.001**
***Friendly/Stimulating/No opinion***	**17**		**11**		**17**		**11**		**18**		**11**	
***Competitive and hostile***	**22**		**11**		**23**		**10**		**22**		**13**	
**Satisfying friendships with classmates**				**<0.001**				**<0.001**				**<0.001**
***Yes/Not yet***	**18**		**10**		**18**		**10**		**18**		**11**	
***No***	**25**		**12**		**24.5**		**13.5**		**24.5**		**10**	
**Feeling that medical school prevents §**												
***Playing sport***				**<0.001**				**<0.001**				**<0.001**
***No***	**17**		**11**		**17**		**11**		**17**		**11**	
***Yes***	**20**		**11**		**20**		**11**		**20**		**12.75**	
***Having hobbies***				**<0.001**				**<0.001**				**<0.001**
***No***	**16**		**10**		**16**		**10**		**16**		**10**	
***Yes***	**20**		**11**		**20**		**11**		**20**		**12**	
***Seeing friends***				**<0.001**				**<0.001**				**<0.001**
***No***	**17**		**10**		**17**		**10**		**17**		**10**	
***Yes***	**20**		**12**		**21**		**12**		**20**		**11.5**	
***Sleeping adequately***				**<0.001**				**<0.001**				**<0.001**
***No***	**16**		**10**		**17**		**11**		**16**		**9**	
***Yes***	**21**		**11**		**21**		**11**		**21**		**11**	
***Resting***				**<0.001**				**<0.001**				**<0.001**
***No***	**16**		**9.75**		**16**		**10**		**16**		**9**	
***Yes***	**20**		**11**		**20**		**11**		**20**		**11**	
**Motives underlying medical school choice §**												
***High gain opportunities***				0.604				0.274				0.794
***No***	18		11		18		11		18		11	
***Yes***	19		12		19		13		18		12	
***Employment opportunities/Social prestige***				0.100				0.605				0.103
***No***	18		11		18		11		19		11	
***Yes***	18		11		18		10		17.5		12	
***Personal/Family history of disease***				**0.006**				**0.074**				**0.033**
***No***	**18**		**11**		**18**		**11**		**18**		**11**	
***Yes***	**19**		**12**		**19**		**12**		**19**		**11.5**	
***Acquaintance/Relative MD influence***				0.416				0.125				0.580
***No***	18		11		18		11		18		11	
***Yes***	17		13		16.5		13.25		19		12	
***Helping those who suffer***				0.555				0.119				0.551
***No***	18		11		19		11		18		11	
***Yes***	18		11		18		11		18		11	
***Interest in human relationships***				0.090				0.229				0.245
***No***	18		11		18		11		18		11	
***Yes***	19		11		19		11		19		11	
***Intellectual curiosity***				0.077				0.027				0.498
***No***	19		11		19		11		18.5		11	
***Yes***	18		11		17		11		18		11	
***Imposition by parents/relatives***				0.086				0.004				0.473
***No***	18		11		18		11		18		11	
***Yes***	22		9.5		26		11.75		17		13	
**Concerns about the future §**												
***No, future is stimulating***				**<0.001**				**<0.001**				**<0.001**
***No***	**20**		**11**		**20**		**11**		**20**		**12**	
***Yes***	**13**		**9**		**13**		**10**		**14**		**9**	
***No, not thinking about future***				**<0.001**				**0.001**				**<0.001**
***No***	**18**		**11**		**18**		**11**		**19**		**11**	
***Yes***	**15**		**8**		**16**		**10**		**14**		**8**	
***Yes, not feeling good enough for the profession***				**<0.001**				**<0.001**				**<0.001**
***No***	**16**		**10**		**16**		**9**		**16**		**10**	
***Yes***	**21**		**11**		**22**		**11**		**21**		**11**	
***Yes, about the specialty choice***				**<0.001**				**<0.001**				**<0.001**
***No***	**17**		**11**		**17**		**11**		**18**		**11**	
***Yes***	**21**		**11**		**21**		**11**		**20.5**		**10**	
***Yes, about limited work/specialty opportunities***				**<0.001**				**<0.001**				**<0.001**
***No***	**16**		**10**		**16**		**10.5**		**16**		**9**	
***Yes***	**21**		**11**		**21**		**11**		**20**		**11**	
**Stimulating drugs**				**0.001**				**0.245**				**<0.001**
***No***	**18**		**11**		**18**		**11**		**18**		**11**	
***Yes***	**20**		**13**		**19.5**		**11.5**		**21**		**12.75**	

^a–c^ Different superscripts if Mann–Whitney U test between groups *p*-value < 0.05 (Bonferroni correction). NLAfH Not Living Away from Home, LAfH Living Away from Home, **§** More than one answer was possible.

**Table 3:** Characteristics associated with perceived stress, stratified by living condition.

Characteristics	All Students			Not Living Away from Home			Living Away from Home		
	B	95% CI	*p*	B	95% CI	*p*	B	95% CI	*p*
**Age 1 yr**	**0.24**	**0.08; 0.39**	**0.003**	0.13	−0.17; 0.42	0.399	**0.27**	**0.08; 0.46**	**0.006**
**Gender**									
***Male***	**Ref.**	**-**	**-**	**Ref.**	**-**	**-**	**Ref.**	**-**	**-**
***Female***	**2.66**	**2.16; 3.16**	**<0.001**	**2.75**	**1.95; 3.54**	**<0.001**	**2.53**	**1.86; 3.20**	**<0.001**
**University location**									
***Northern Italy***	**Ref.**	**-**	**-**	**Ref.**	**-**	**-**	**Ref.**	**-**	**-**
***Central Italy***	**1.00**	**0.38; 1.63**	**0.002**	**1.18**	**0.18; 2.19**	**0.021**	**0.86**	**0.04; 1.68**	**0.039**
***Southern Italy***	**0.95**	**0.40; 1.49**	**<0.001**	**1.24**	**0.39; 2.09**	**0.004**	0.67	−0.05; 1.40	0.070
**Year of university**									
***1st***	**Ref.**	**-**	**-**	Ref.	-	-	**Ref.**	**-**	**-**
***4th***	−0.11	−0.88; 0.65	0.772	0.84	−0.49; 2.17	0.214	−0.70	−1.67; 0.27	0.160
***6th***	**−1.55**	**−2.59; −0.50**	**0.004**	−0.19	−2.08; 1.70	0.843	**−2.36**	**−3.67; −1.05**	**<0.001**
**Chronic disease †**	**1.03**	**0.11; 1.95**	**0.029**	0.56	−0.94; 2.06	0.465	**1.26**	**0.05; 2.46**	**0.041**
**Currently in a relationship**									
***Single***	**Ref.**	**-**	**-**	**Ref.**	**-**	**-**	Ref.	-	-
***Involved***	**−0.71**	**−1.19; −0.23**	**0.004**	**−1.02**	**−1.78; −0.25**	**0.009**	−0.5	−1.12; 0.13	0.117
**Being part of a sexual minority**									
***No***	**Ref.**	**-**	**-**	**Ref.**	**-**	**-**	**Ref.**	**-**	**-**
***Yes***	**1.44**	**0.74; 2.13**	**<0.001**	**2.14**	**1.07; 3.20**	**<0.001**	**1.08**	**0.14; 2.03**	**0.025**
**Family history of mental illness †**	**0.65**	**0.07; 1.23**	**0.027**	0.31	−0.62; 1.23	0.514	**0.84**	**0.08; 1.59**	**0.030**
**Family history of suicide †**	**1.22**	**0.18; 2.26**	**0.022**	1.40	−0.30; 3.11	0.107	1.04	−0.30; 2.37	0.128
**Family relationship evaluation**									
***Poor/Excessive***	**Ref.**	**-**	**-**	**Ref.**	**-**	**-**	**Ref.**	**-**	**-**
***Good***	**−0.89**	**−1.73; −0.05**	**0.037**	**−1.55**	**−2.81; −0.30**	**0.015**	−0.37	−1.53; 0.79	0.536
***Excellent***	**−2.17**	**−3.00; −1.35**	**<0.001**	**−2.93**	**−4.18; −1.68**	**<0.001**	**−1.72**	**−2.86; −0.58**	**0.003**
**Financial situation**									
***Good***	**Ref.**	**-**	**-**	**Ref.**	**-**	**-**	**Ref.**	**-**	**-**
***Poor***	**2.59**	**1.75; 3.43**	**<0.001**	**2.94**	**1.63; 4.25**	**<0.001**	**2.33**	**1.21; 3.45**	**<0.001**
**Working because of need †**	0.80	−0.55; 2.15	0.245	1.04	−0.88; 2.97	0.287	0.42	−1.53; 2.37	0.673
**Having a hobby †**	**−1.36**	**−1.89; −0.82**	**<0.001**	**−1.27**	**−2.11; −0.42**	**0.003**	**−1.57**	**−2.27; −0.87**	**<0.001**
**Playing sport**									
***No***	Ref.	-	-	Ref.	-	-	Ref.	-	-
***Less than 90′ a week***	−0.58	−1.17; 0.00	0.051	−0.41	−1.34; 0.52	0.387	−0.58	−1.37; 0.20	0.147
***More than 90′ a week***	−0.46	−1.09; 0.17	0.149	−0.35	−1.43; 0.72	0.520	−0.43	−1.22; 0.37	0.293
**Current opinion on the medical school choice**									
***Positive***	**Ref.**	**-**	**-**	**Ref.**	**-**	**-**	**Ref.**	**-**	**-**
***Negative/No opinion***	**3.00**	**3.63; 2.37**	**<0.001**	**3.17**	**4.22; 2.12**	**<0.001**	**2.81**	**3.61; 2.00**	**<0.001**
**Current opinion on medical class atmosphere**									
***Friendly/Stimulating/No opinion***	**Ref.**	**-**	**-**	**Ref.**	**-**	**-**	**Ref.**	**-**	**-**
***Competitive and hostile***	**1.80**	**1.10; 2.49**	**<0.001**	**1.88**	**0.75; 3.00**	**0.001**	**1.83**	**0.92; 2.73**	**<0.001**
**Satisfying friendships with classmates**									
***Yes/Not yet***	**Ref.**	**-**	**-**	**Ref.**	**-**	**-**	**Ref.**	**-**	**-**
***No***	**2.46**	**1.30; 3.62**	**<0.001**	**1.13**	**−0.76; 3.01**	**0.241**	**3.03**	**1.53; 4.53**	**<0.001**
**Feeling that medical school prevents ‡**									
***Having hobbies***	**0.82**	**0.30; 1.34**	**0.002**	**0.80**	**−0.03; 1.64**	**0.060**	**0.79**	**0.11; 1.47**	**0.023**
***Sleeping adequately***	**1.91**	**1.38; 2.44**	**<0.001**	**1.09**	**0.27; 1.91**	**0.009**	**2.53**	**1.83; 3.23**	**<0.001**
***Resting***	**1.10**	**0.58; 1.61**	**<0.001**	**0.97**	**0.15; 1.79**	**0.020**	**1.20**	**0.52; 1.88**	**<0.001**
**Motives underlying medical school choice ‡**									
***Intellectual curiosity***	**−1.09**	**−1.57; −0.61**	**<0.001**	**−1.12**	**−1.88; −0.36**	**0.004**	**−1.11**	**−1.74; −0.49**	**<0.001**
**Concerns about the future ‡**									
***No, future is stimulating***	**−1.74**	**−2.47; −1.02**	**<0.001**	**−2.40**	**−3.57; −1.23**	**<0.001**	**−1.17**	**−2.12; −0.21**	**0.017**
***Yes, not feeling good enough for the profession***	**2.46**	**1.89; 3.02**	**<0.001**	**2.49**	**1.58; 3.40**	**<0.001**	**2.50**	**1.77; 3.24**	**<0.001**
***Yes, about the specialty choice***	**0.92**	**0.30; 1.53**	**0.004**	**0.45**	**−0.52; 1.43**	**0.363**	**1.21**	**0.40; 2.02**	**0.004**
***Yes, about limited work/specialty opportunities***	**1.01**	**0.44; 1.58**	**<0.001**	**0.66**	**−0.24; 1.56**	**0.153**	**1.29**	**0.54; 2.04**	**<0.001**

B = Unstandardized B coefficients, Ref. = Reference category. † Yes/no answers. Yes answer coefficients are reported. ‡ More than one answer was possible.

## Data Availability

The data that support the findings of this study are available on request from the corresponding author. The data are not publicly available due to privacy restrictions.

## Supplementary Materials

The following supporting information can be downloaded at: https://www.mdpi.com/article/10.3390/ijerph19095010/s1. The socio-demographic questionnaire used in the study (translated in English) is provided.
