# Supplementary material for: Stress in Medical Students: PRIMES, an Italian, Multicenter Cross-Sectional Study
Source: Int J Environ Res Public Health. 2022 Apr 20;19(9):5010. doi: 10.3390/ijerph19095010 (PMC9100187; doi:10.3390/ijerph19095010)
Supplement: Supplementary file 1 [file ijerph-19-05010-s001.zip › ijerph-1669604-supplementary.pdf]

PRIMES sociodemographic questionnaire (translated version)

**1. Gender**:	❑ Male<br>❑ Female<br>❑ Not binary/third gender	**2. Age:**	|__|__| years
**3. Nationality:**	❑ Italian<br>❑ Not Italian: ____________	**4. Are you studying far from home?**	❑ No<br>❑ Yes, but I am from the same region of my University<br>❑ Yes, I am from a different region than my University one. Specify which region: ______________<br>❑ Yes, I am from another country Specify which country: __________________________

5. At present, who do you live with?
- ❑ Alone
- ❑ With parents
- ❑ With relatives
- ❑ With partner
- ❑ With housemates
- ❑ In a dormitory

6. How do you think the cohesion of your family is?
- ❑ Very poor
- ❑ Poor
- ❑ Good
- ❑ Excellent
- ❑ Excessive

7. Relationship status:
- ❑ Single
- ❑ Involved

8. At present, who do you feel sexually attracted to?
- ❑ Only men
- ❑ Mainly men
- ❑ Both men and women in the same way
- ❑ Mainly women
- ❑ Only women
- ❑ Neither men nor women

9. Do you have first/second degree relatives with diagnosed psychiatric illnesses?
- ❑ No
- ❑ Yes. How many? |__|__|
  With what disease? (*you can select more than one answer*)
  - ❑ Schizophrenia spectrum and other psychotic disorders
  - ❑ Bipolar disorders
  - ❑ Depressive disorders
  - ❑ Anxiety disorders
  - ❑ Obsessive-compulsive disorders
  - ❑ Feeding and eating disorders
  - ❑ Substance-related and addictive disorders
  - ❑ Personality disorders
  - ❑ Other: ____________________

10. Were there suicides and/or suicide attempts in your family (including only first/second degree relatives)?
- ❑ No
- ❑ Yes. How many? |__|__|

11. Do you suffer from a chronic disease?
- ❑ No
- ❑ Yes: _____________________

12. How would you rate your family's financial situation with respect to your needs?
- ❑ Insufficient
- ❑ Poor
- ❑ Adequate
- ❑ Excellent

13. Do you have a job?

- ❑ No
- ❑ Yes, by necessity and I can almost/fully provide for myself
- ❑ Yes, by necessity but I can't provide for myself
- ❑ Yes, but not by necessity

14. Do you exercise?

- ❑ Yes, more than 90 min per week
- ❑ Yes, less than 90 min per week
- ❑ Yes, occasionally, less than once a week
- ❑ No

15. Do you have a personal passion/hobby in your spare time?

- ❑ Yes
- ❑ No

16. Do you think that medical school prevents you from..?(*you can select more than one answer*)

- ❑ I don't think that Medical School prevents me from doing anything
- ❑ Exercising as I wish
- ❑ Having hobbies as I wish
- ❑ Seeing friends as I wish
- ❑ Sleeping properly (quantitatively and/or qualitatively)
- ❑ Resting and relaxing as I wish

17. Did you choose medical school mainly for..? (*you can select more than one answer*)

- ❑ High gain opportunities
- ❑ Employment opportunities and social status
- ❑ Personal/family experience of disease
- ❑ Influence by an acquaintance/relative who is a medical doctor
- ❑ Helping people who suffer
- ❑ Interest in human relations
- ❑ Intellectual curiosity
- ❑ Imposition by parents/relatives

18. At present, how do you judge the choice made?

- ❑ Positively
- ❑ Negatively
- ❑ I don't know

19. Year of course that you are attending: ______ (***If you are at 1st year, go to question n. 23***)

20. In which range do you place the arithmetic average of grades of the exams you took?

- ❑ 18-20.99
- ❑ 21-23.99
- ❑ 24-26.99
- ❑ 27-28.99
- ❑ ≥29

21. Are your satisfied with your grade average?

- ❑ Yes
- ❑ No, but it's not a problem. Grade average is not my priority
- ❑ No, I want to work harder to improve
- ❑ No, but I am already working hard and I don't think my grade average reflects my diligence

22. Are you in time with the exams?

- ❑ Yes
- ❑ No, but it's not a problem
- ❑ No and it's a problem

23. Is there a psychological counselling in your university?

- ❑ Yes
- ❑ No
- ❑ I don't know

24. Would you use it in case of need?

- ❑ Yes
- ❑ No
- ❑ I don't know

25. How would you define the climate between your classmates?
(Choose only one answer that you think best suits you)

- ❑ Friendly and relaxed
- ❑ Competitive and stimulating
- ❑ Competitive and hostile
- ❑ I don't have an opinion yet

26. Have you built friendships with a circle of classmates that are satisfying for you?

- ❑ Yes
- ❑ Not yet, but I wish I will
- ❑ No, I don't feel the need
- ❑ No, I don't think that the friendships that I built are satisfying

27. Do you have any worries about the future? (*you can select more than one answer*)

- ❑ No, I think that the future is stimulating
- ❑ No, I am not thinking about my future at the moment
- ❑ Yes, I am worried about not measuring up to the profession
- ❑ Yes, I am worried about the choice of the specialty
- ❑ Yes, I am worried about the limited number of places for specialty/job

28. Have you ever taken stimulants to improve your academic performance (exams, internships)?

- ❑ No
- ❑ Yes. Which? (exclude coffee/energizing drinks) __________________

29. Have you ever taken psychiatric medications during you university career?

- ❑ No
- ❑ Yes. Complete the following table by writing the name of the medication(s) in the space with dots and placing a cross on the box indicating the respective frequency of use:

Name of medication:	Rarely	Monthly	Weekly	Daily
…………………..				
…………………..				
…………………..				
…………………..				

30. Are you seeing a psychologist/psychiatrist?

- ❑ Yes
- ❑ No
